# OP72 Patient Organization Submissions Made To A National Health Technology Assessment Agency In Ireland

**DOI:** 10.1017/S0266462324001314

**Published:** 2025-01-07

**Authors:** Joan O’Callaghan, Lesley Tilson, Roisin Adams, Laura McCullagh

## Abstract

**Introduction:**

The National Centre for Pharmacoeconomics (NCPE) has a Patient Organisation Submission Process (POSP) enabling patients to communicate their experiences to the drug-reimbursement decision-maker. The NCPE proactively invites and supports patient organizations to make submissions to the decision-maker for ongoing health technology assessments (HTAs). We evaluate uptake of the POSP and determine whether submission trends differ by drug type.

**Methods:**

We reviewed all HTAs completed by the NCPE since 2016 (when the POSP was first introduced) to present (data cut-off date 22 November 2023). Time trends in the proportions of HTAs for which a patient organization submission had been made to the NCPE were analyzed descriptively. We also compared the proportions of HTAs for which a patient organization submission had been made for (i) orphan versus non-orphan drugs and (ii) oncology versus non-oncology drugs.

**Results:**

The number of patient organization submissions made to the NCPE has increased over time. In 2016, 24 percent (6/25) of completed HTAs were associated with a patient organization submission compared with 50 percent (11/22) in 2023. The proportions of completed HTAs associated with a patient organization submission are comparable between orphan (38%; 24/64) and non-orphan drugs (29%; 34/117) (Chi²; p=0.245). The proportion of completed HTAs for which a patient organization submission had been made was lower for oncology drugs (14%; 14/97) versus non-oncology drugs (52%; 44/84) (Chi²; p<0.001).

**Conclusions:**

Patient organization submissions to the NCPE have increased over time. The proportions of HTAs for which patient organization submissions have been made are comparable between orphan and non-orphan drugs. However, the proportion of patient organization submissions for oncology drugs is lower than the proportion for non-oncology drugs. The NCPE will continue to liaise with patient organizations to increase engagement with the POSP.

